# Lethal effects of mitochondria via microfluidics

**DOI:** 10.1002/btm2.10461

**Published:** 2022-12-05

**Authors:** Hyueyun Kim, Young‐Ho Ahn, Chang Mo Moon, Jihee Lee Kang, Minna Woo, Minsuk Kim

**Affiliations:** ^1^ Department of Pharmacology College of Medicine, Ewha Womans University Seoul Republic of Korea; ^2^ Department of Molecular Medicine College of Medicine, Ewha Womans University Seoul Republic of Korea; ^3^ Department of Internal Medicine College of Medicine, Ewha Womans University Seoul Republic of Korea; ^4^ Department of Physiology and Inflammation‐Cancer Microenvironment Research Center College of Medicine, Ewha Womans University Seoul Republic of Korea; ^5^ Division of Endocrinology and Metabolism, Department of Medicine Toronto General Hospital, Research Institute, University Health Network, University of Toronto Toronto Ontario Canada

**Keywords:** breast tumor cell, cotton candy, microfluidics, mitochondria, refractive index, tomographic microscope, tunneling nanotube

## Abstract

Tumor cells can respond to therapeutic agents by morphologic alternations including formation of tunneling nanotubes. Using tomographic microscope, which can detect the internal structure of cells, we found that mitochondria within breast tumor cells migrate to an adjacent tumor cell through a tunneling nanotube. To investigate the relationship between mitochondria and tunneling nanotubes, mitochondria were passed through a microfluidic device that mimick tunneling nanotubes. Mitochondria, through the microfluidic device, released endonuclease G (Endo G) into adjacent tumor cells, which we referred to herein as unsealed mitochondria. Although unsealed mitochondria did not induce cell death by themselves, they induced apoptosis of tumor cells in response to caspase‐3. Importantly, Endo G‐depleted mitochondria were ineffective as lethal agents. Moreover, unsealed mitochondria had synergistic apoptotic effects with doxorubicin in further increasing tumor cell death. Thus, we show that the mitochondria of microfluidics can provide novel strategies toward tumor cell death.

## 1 INTRODUCTION

Tumor cell can undergo various morphological changes in response to different therapeutic agents. For example, mammalian target of rapamycin (mTOR) can convert tumor cells into fat‐like cells.^1^, ^2^, ^3^, ^4^ Various morphological alterations in lamellipodia, filopodia, or tunneling nanotubes have been shown to occur in these cells.^5^, ^6^ However, the functional significance of tunneling nanotubes during tumor cell reprogramming has not been fully elucidated.

Tunneling nanotubes are thin membrane elongations that are formed between cells that can mediate trafficking of subcellular vesicles, proteins, or organelles.^7^ They consist of filamentous‐actin and are about 0.05–1 μm in width and 100 μm in length.^7^ In addition, they have been implicated as one mechanism by which mitochondria can be transferred from one cell to another.^8^ The speed of mitochondrial migration through tunneling nanotubes is about 80–1400 nm/s.^9^ Several studies have been conducted on mitochondrial migration between cells.^10^, ^11^, ^12^, ^13^ An in vitro study showed that oxidative stress‐induced apoptosis of endothelial cells or H9c2 cardiomyocytes was abolished by transferring of mitochondria from mesenchymal stem cells.^14^, ^15^ An in vivo study showed that mitochondrial transfer inhibited hypoxia‐induced apoptosis in cardiomyocytes.^16^ Moreover, mitochondria can be transferred to support the survival of metabolically compromised cells.^17^, ^18^ Therefore, mitochondrial transfer through tunneling nanotubes can provide important insight in our understanding of tumor cell fate.

We describe here the application of optical tomography whereby an imaging technique is used to obtain cross sections of cells remotely.^19^ In optical tomography, projected images are obtained by waves passing through the cells at various angles, and digital images are subsequently reconstructed to obtain the internal structure of cells in a cross‐sectional manner.^19^, ^20^ Since the tomographic microscope can render reconstructed cell images according to varying refractive index, transparent objects such as lipid droplets and mitochondria can be detected without staining.^21^, ^22^ Here, we examined breast tumor cells and their response to antitumor agents, rho‐associated protein kinase (ROCK) and mTOR inhibitors by tomographic microscopy. Intriguingly, we found that mitochondria within tumor cells transferred into other nearby tumor cells via tunneling nanotubes and induced apoptosis.

Therefore, we hypothesized that mitochondria of tunneling nanotubes can be an effective novel strategy to achieve tumor cell death. To this end, we investigated the mechanisms by which mitochondria can instigate tumor apoptosis. Specifically, we examined the physical processing of mitochondria that occurs during transfer through tunneling nanotubes and recapitulation of this process.

## 2 MATERIALS AND METHODS

### 2.1 Animal treatment

All mice were in C57BL/6 background and purchased from Charles River. All experiments using animals were performed and approved by the NIH guidelines (Guide for the care and use of laboratory animals) and the Ewha Womans University Animal Care Committee respectively. Forty female mice were each given 6 weekly 1 mg doses of 7,12‐dimethylbenzathracene (DMBA) in 0.2 ml of sesame oil by oral gavage and implanted 30 mg of subcutaneous pellets of compressed medroxyprogesterone acetate (MPA), beginning at 5 weeks of age. Doxorubicin was administered weekly by intraperitoneal injection (5 mg/kg body weight, 300 μl) for 4 weeks. Mice were then maintained for another week.

### 2.2 Cell culture

Human breast cancer cell lines, MDA‐MB‐453, MCF‐7, and MDA‐MB‐231 (ATCC, USA), were maintained in Dulbecco's MEM (11885; Gibco, USA) with 10% fetal bovine serum (16000044; Gibco). Human neonatal cardiomyocytes (36044‐21; Celprogen, USA) were cultured in cardiomyocyte media (M36044‐21; Celprogen). All cultures were maintained at 37°C under an atmosphere of 95% O_2_ and 5% CO_2_.

### 2.3 Breast tumor cell reprogramming

Breast tumor cells were plated at a density of 1.5 × 10^4^ cells in 35 mm dishes. For adipocyte induction, cells were incubated with ROCK–mTOR inhibitors and differentiation inducing chemical cocktail as follows: 2 μM Y27632 (1254; Tocris, USA), 2 μM rapamycin (1292; Tocris), 1 μM dexamethasone (1126; Tocris), 0.5 μM isobutylmethylxanthine (I5879; Sigma‐Aldrich, USA), and 200 μM indomethacin (I7378; Sigma‐Aldrich). Cells were grown under these conditions for up to 4 days. Adipocyte differentiation was visualized with refractive index using tomographic microscope.

### 2.4 Optical tomographic microscope

Green light (*λ* = 520 nm, exposure 0.2 mw/mm^2^) from a laser diode was split into cells and reference beam at Nanolive (3D cell explorer, Switzerland). Cells were illuminated with a laser beam inclined at 45°, which rotates around the sample 360°. Holographic images were recorded on a digital camera by combining the beam that had passed through the cells with the reference beam. 3D cell images were recorded up to 30 μm depth of reconstruction. The fluorophore Mitotracker green (ex/em maxima ~490/516; Life Technologies, M7514) was used to detect mitochondrial movement. Prior to each experiment, cells were stained with 50 nM Mitotracker green for 15 min.

### 2.5 Quantitation of fat‐like cells

We defined fat‐like cells when the sum of the area of the lipid droplets accounted for about 30% of the total cell area. At the start of the experiment, the number of MDA‐MB‐453 was approximately 10^4^ in a 2 cm‐diameter dish. Following treatment with ROCK–mTOR inhibitors, approximately 3 × 10^3^ (30%) of cells were dead, 2.5 × 10^3^ (25%) were nondifferentiated, and 4.5 × 10^3^ (45%) of cells were differentiated into fat‐like cells. For each experiment, three triplicate plates were used to obtain an average value, and the experiments were repeated six different times.

### 2.6 Experimental procedures using microfluidic device

Microfluidic device with a diameter of 300 μm was purchased from Microfit. Cotton candy (CC) sheets were sealed with polydimethylsiloxane (PDMS) to make microfluidic mold. After hardening, CC fibers were removed by perfusing with water to make microfluidic mold of approximately 1 μm. Each microfluidic device was connected by polythene tubing (PE10; Braintree Scientific, USA) with an inner diameter of 0.28 mm. Prior to each experiment, isopropanol (W292907; Sigma‐Aldrich) was flushed through the microfluidic channel to remove all air bubbles followed by 1X PBS (10010023; Gibco, USA) wash for 30 min. Fluid flow was controlled by individual peristaltic pump (3200243, Dolomite, UK). Isolated mitochondria were introduced to microfluidic devices at a flow rate of 10–30 μm/s. After passing through the microfluidic channels, mitochondria were transported into cells.

### 2.7 Analysis of mRNA using real‐time PCR

mRNA levels of *ADD1* (Hs05044793_s1), *ADIPOQ* (Hs00605917_m1), *FABP* (Hs00155026_m1), *FIP1L1* (Hs01547450_m1), *PAK2* (Hs02559219_s1), *PLIN1* (Hs00160173_m1), *PPARG* (Hs01115513_m1), *RARRES2* (Hs00414615_g1), and *ENDOG* (Hs00172770_m1) were determined using primer/probe set from Life Technology. Real‐time PCR was performed with TaqMan universal PCR Master Mix on an ABI Real time PCR System 7000 (Applied Biosystems, USA). PCR conditions were 50°C for 2 min and 95°C for 10 min, followed by 40 cycles of 95°C for 15 s and 60°C for 1 min. For each experimental sample, the relative abundance value was normalized to the value derived from *ACTB* (Hs03023943_g1; Life Technology) as housekeeping control gene. Relative mRNA levels were quantified using the comparative 2^−ΔΔCT^ method.

### 2.8 ELISA for detection of apoptosis

Apoptosis was determined using ssDNA ELISA Kit (APT225; Sigma‐Aldrich). Briefly, cells were incubated with rapamycin and Y27632 in 96 well plates up to 4 days. Cell plates were fixed and incubated with ABTS solution for 30 min to allow binding to HRP at 37°C. To denature DNA, cell plates were incubated for 20 min at 75°C. After cooling at 4°C for 5 min, plates were blocked in 5% skim milk (70166; Sigma‐Aldrich) in PBS at 37°C for 1 h. Cells were incubated with antisera mixture for 30 min and washed with PBS. After treatment with stop solution, absorbance was measured at 405 nm.

### 2.9 ELISA for Caspase‐3

Caspase‐3 activity was determined using Cleaved Caspase‐3 ELISA kit (ab220655; abcam, USA). Cells were fixed and incubated with antibody cocktail at 37°C for 1 h, then washed and incubated with tetramethylbenzidine (TMB) solution for 30 min. After treatment with stop solution, absorbance was measured at 450 nm.

### 2.10 Western blot

Briefly, MDA‐MB‐453, MCF‐7, and MDA‐MB‐231 cell lines or neonatal cardiomyocytes were homogenized in ice‐cold lysis buffer. After centrifugation at 5000*g* for 20 min, protein content of the supernatant was quantified using the Bradford protein assay. Samples were diluted, boiled with sample loading dye, and 100 μg were loaded in SDS‐PAGE (4561033EDU; Bio‐Rad). After blotting, membranes were blocked in 5% skim milk (70166; Sigma‐Aldrich) in PBS containing 0.1% Tween‐20 (P1379; Sigma‐Aldrich). Membranes were incubated with antisera directed against cytochrome C (1:1000; #11940, Cell Signaling Technology, USA), Endo G (1:1000; #4969, Cell Signaling Technology), or AIF (1:1000; ab1998, Abcam, USA), then with secondary antibodies (mouse‐specific HRP‐conjugated antibody or rabbit‐specific HRP‐conjugated antibody). Bands were visualized using ECL (32106, Thermo Scientific) detection kit and quantified by densitometry. Blots were stripped and re‐exposed to detect β‐actin (1:1000; sc‐47778, Santacruz Biotechnology, USA) as housekeeping protein.

### 2.11 Mitochondrial isolation and transfer

Cells were harvested with homogenization buffer (20 mM HEPES‐KOH (pH 7.4), 220 mM mannitol, and 70 mM sucrose) containing a protease inhibitor mixture (Sigma‐Aldrich) and centrifuged at 2300*g* for 5 min. The cell pellet was resuspended with homogenization buffer and incubated on ice for 5 min at 4°C. Cells were ruptured by 10 strokes using a 27‐gauge needle. The homogenate was centrifuged at 400*g* for 5 min. After centrifugation at 5800*g* for 5 min, mitochondria were harvested and suspended with homogenization buffer. The amount of isolated mitochondria was expressed as protein concentration by using the Bio‐Rad protein assay kit (Bio‐Rad, Richmond, USA). Mitochondrial transfer was conducted by co‐incubating isolated mitochondria with cells (1 × 10^5^ cells/well of a six‐well plate) at 37°C under 5% CO_2_ for up to 4 days. Stained mitochondria were treated daily for up to 4 days. For in vivo experiments, mice were administered weekly with 1 × 10^4^ isolated mitochondria per gram of body weight via the tail vein.

### 2.12 Transmission electron microscopy

We fixed the mold of CC‐microfluidic with 3% buffered glutaraldehyde (G5882; Sigma‐Aldrich) for 2 h and processed into resin (02334; Polysciences, German). After embedding, the resin block was thin‐sectioned by ultramicrotomy. Sections of 50–70 nm thickness were collected on metal mesh and stained with electron dense particles before imaging of ultrastructures, using the transmission electron microscope (H‐7650; Hitachi‐Science & Technology, Japan).

### 2.13 CRISPR‐mediated gene deletion

Clustered regularly interspaced short palindromic repeats (CRISPR) transfection of *ENDOG* in MDA‐MB‐453 was performed using a kit from Santa Cruz (sc‐395739; Santacruz Biotechnology, USA). Briefly, in six‐well plates, 10^6^ cells were plated and exposed to the *ENDOG* plasmid (sc‐403263, Santacruz Biotechnology) or negative control‐CRISPR plasmid (sc‐418922; Santacruz Biotechnology, USA) solution for 8 h at 37°C in a CO_2_ incubator. Then, the media was changed to Dulbecco's MEM with 10% fetal bovine serum and incubated for another 18 h. The *ENDOG* expression was determined using RT‐PCR.

### 2.14 Echocardiographic assessment

For echocardiography, Vevo 2100 was used at Cardiovascular Research Center in Seoul, Korea. Mice were anesthetized with 2% isoflurane and maintained with 1.5% isoflurane followed by application of depilatory cream to the chest and wiped clean to remove all hair in the area of interest. The scanning probe (20 MHz) was used to obtain 2D images of the parasternal long axis. These 2D images were converted to M‐mode.

### 2.15 Statistical analysis

Values were means ± SE. The significance of differences was determined by a two‐way analysis of variance (ANOVA), or a one way ANOVA followed by a Bonferroni post hoc analysis where appropriate. Tukey's hsd (honestly significant difference) test was used to confirm synergistic effect, supported by department of statistics at Ewha Womans University. Differences were considered significant when *p* < 0.05.

## 3 RESULTS

### 3.1 Reprogramming of breast tumor cells into fat‐like cells

**FIGURE 1:**
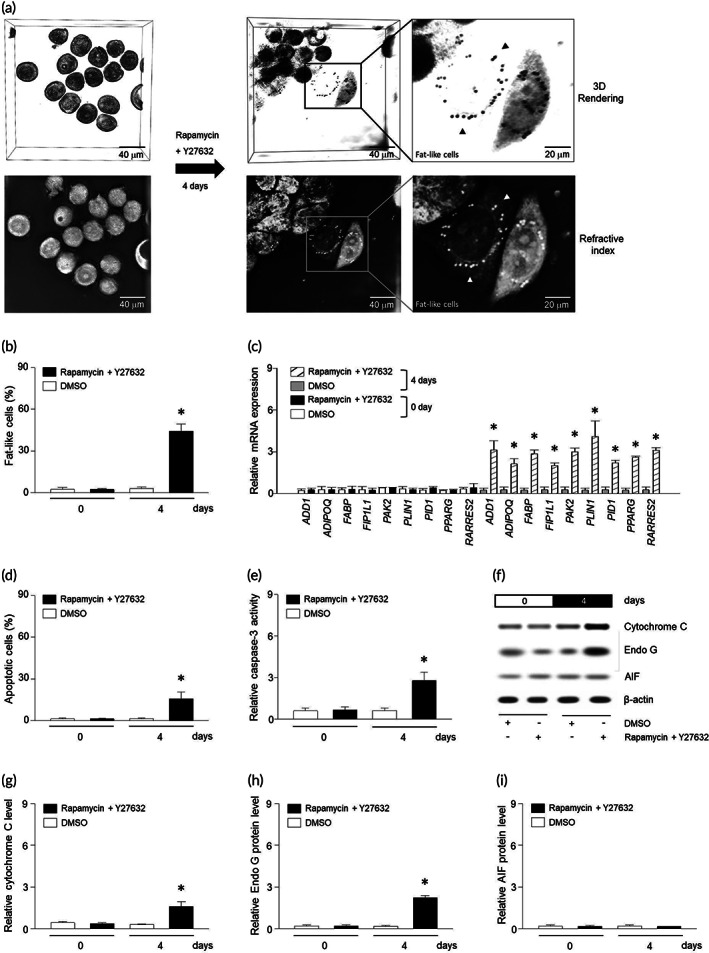
Rho‐associated protein kinase (ROCK)–mammalian target of rapamycin (mTOR) inhibitors induce differentiation of breast tumor cells. (a) Following treatment of rapamycin and Y27632, MDA‐MB‐453 cells were observed by tomographic microscopy and expressed with 3D rendering and refractive index for 4 days. Lipid droplets were clearly distinguished with tomographic images. (b) Quantization of fat‐like cells was counted by lipid droplets. (c) qRT‐PCR results show relative mRNA levels of defined fat differentiation factors. Apoptosis (d) and caspase‐3 (e) were determined using ELISA kit. (f–i) Proteins were extracted from MDA‐MB‐453 cells and expression of Cytochrome C, Endo G, or AIF was determined using western blotting. Lipid droplets are indicated by arrow heads. Results are the means ± SE of six experiments in each group. Three replicates were performed and the average value was obtained. And then the experiments were repeated on 6 different days. *Significantly different from treatment of rapamycin and Y27632 for 0 days, *p* < 0.05

It has been known that breast tumor cells can differentiate into fat‐like cells with ROCK–mTOR inhibitors.^4^ This process was scanned with a *λ* = 520 nm laser beam of tomographic microscopy, and the holographic images were recorded and demonstrated as 3D Rendering and Refractive index difference (Figure 1a). On the 4th day, lipid droplets were clearly observed at refractive index images of MDA‐MB‐453 cells (Figure 1a,b) and mRNA levels of the adipocyte markers (*ADD1*, *ADIPOQ*, *FABP*, *FIP1L1*, *PAK2*, *PLIN1*, *PID1*, or *PPARG*, *RARRES2*) were increased following ROCK–mTOR inhibitors (Figure 1c). ELISA to detect ssDNA showed that 25% of tumor cells were apoptotic in response to ROCK–mTOR inhibitors (Figure 1d). In addition, levels of cleaved caspase‐3, cytochrome C, and Endo G but not apoptosis‐inducing factor (AIF) were elevated in the cytosol (Figure 1e–i).

### 3.2 Detection of tunneling nanotube using tomographic microscopy

**FIGURE 2:**
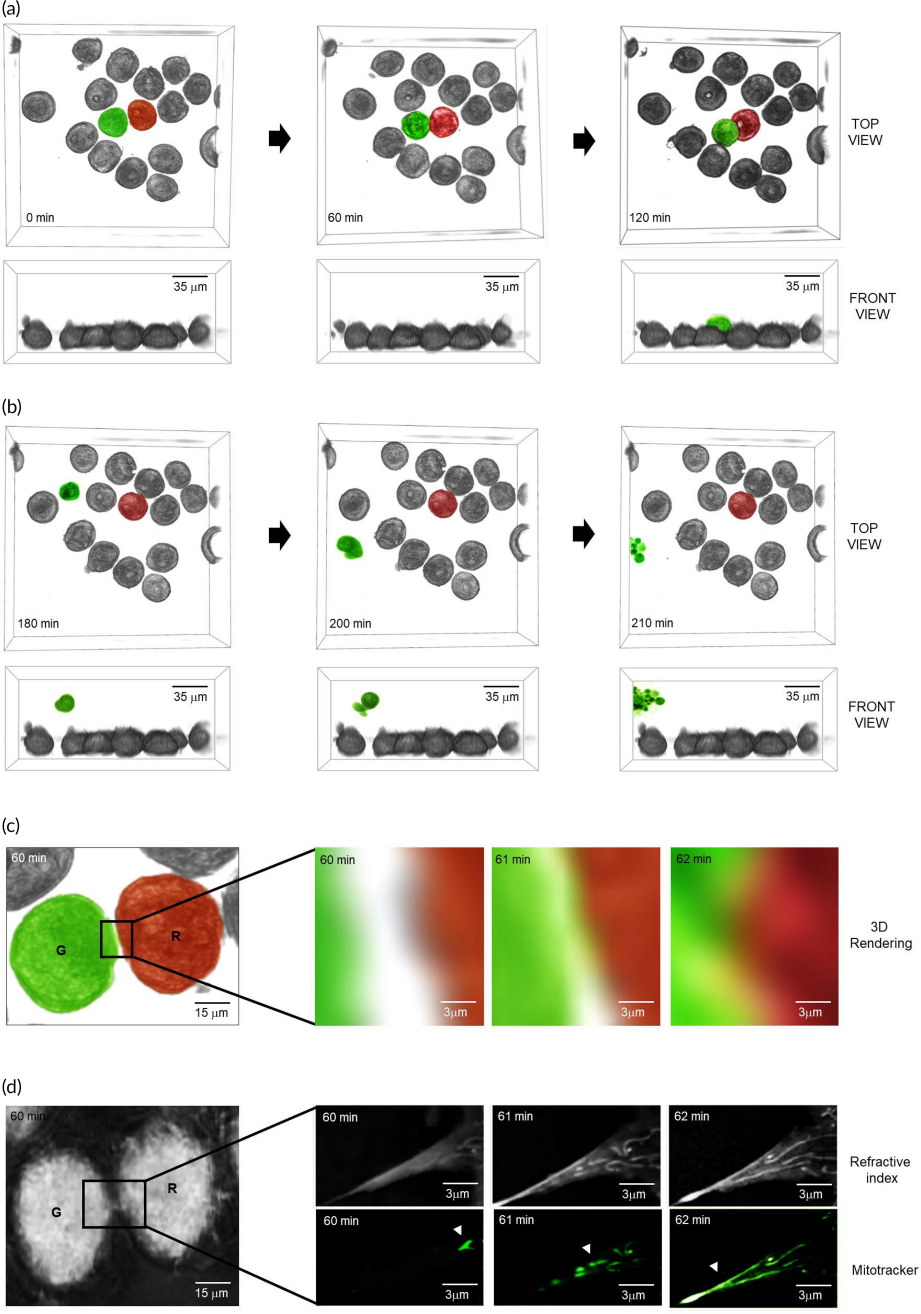
3D rendered images of MDA‐MB‐453. (a) Cell images were rendered with top views and front views by tomographic microscopy at 0, 60, and 120 min. Adjacent cells were digitally stained in green or red. (b) Cell shrinkage or membrane blebbing was observed at 180, 200, and 210 min. (c) 3D rendering images were enlarged at 60, 61, and 62 min. (d) Mitochondria were stained and visualized with mitotracker at 490 nm. Refractive index images were enlarged at 60, 61, and 62 min. Mitochondria are indicated by arrow heads.

Following treatment with ROCK–mTOR inhibitors, 3D‐rendered images of MDA‐MB‐453 cells were obtained in top view and front view (Figure 2a,b). For easy identification, adjacent cells were digitally colored in green or red. At 120 min of the treatment, the green‐colored cell migrated over the red‐stained cells (Figure 2a). At 180 min, the green‐colored cells were detached and suspended in the cell media, resulting in various morphological alterations including cell shrinkage and membrane blebbing (Figure 2b), which are classical morphological features of cells undergoing apoptosis. In order to capture higher resolution changes, 3D‐rendered and refractive index images were further enlarged, whereby we detected red and green cells migrating in closer proximity (Figure 2c). Intriguingly, we observed that the tubular‐shaped parts moved from the red to green cell in the magnified refractive index image (Figure 2d). Mitotracker staining confirmed that these were mitochondria (Figure 2d). With L‐778123 treatment, tunneling nanotubes were not formed and mitochondria were not able to migrate even to cells in close proximity (Figure S1A). In addition, L‐778123 decreased apoptosis and Endo G levels in response to rapamycin and Y27632 (Figure S1B,C). However, L‐778123 did not affect caspase‐3 activity following rapamycin and Y27632 treatment (Figure S1D). Therefore, mitochondrial tunneling preceded apoptosis in this setting.

### 3.3 Internalization of isolated mitochondria in breast tumor cells

**FIGURE 3:**
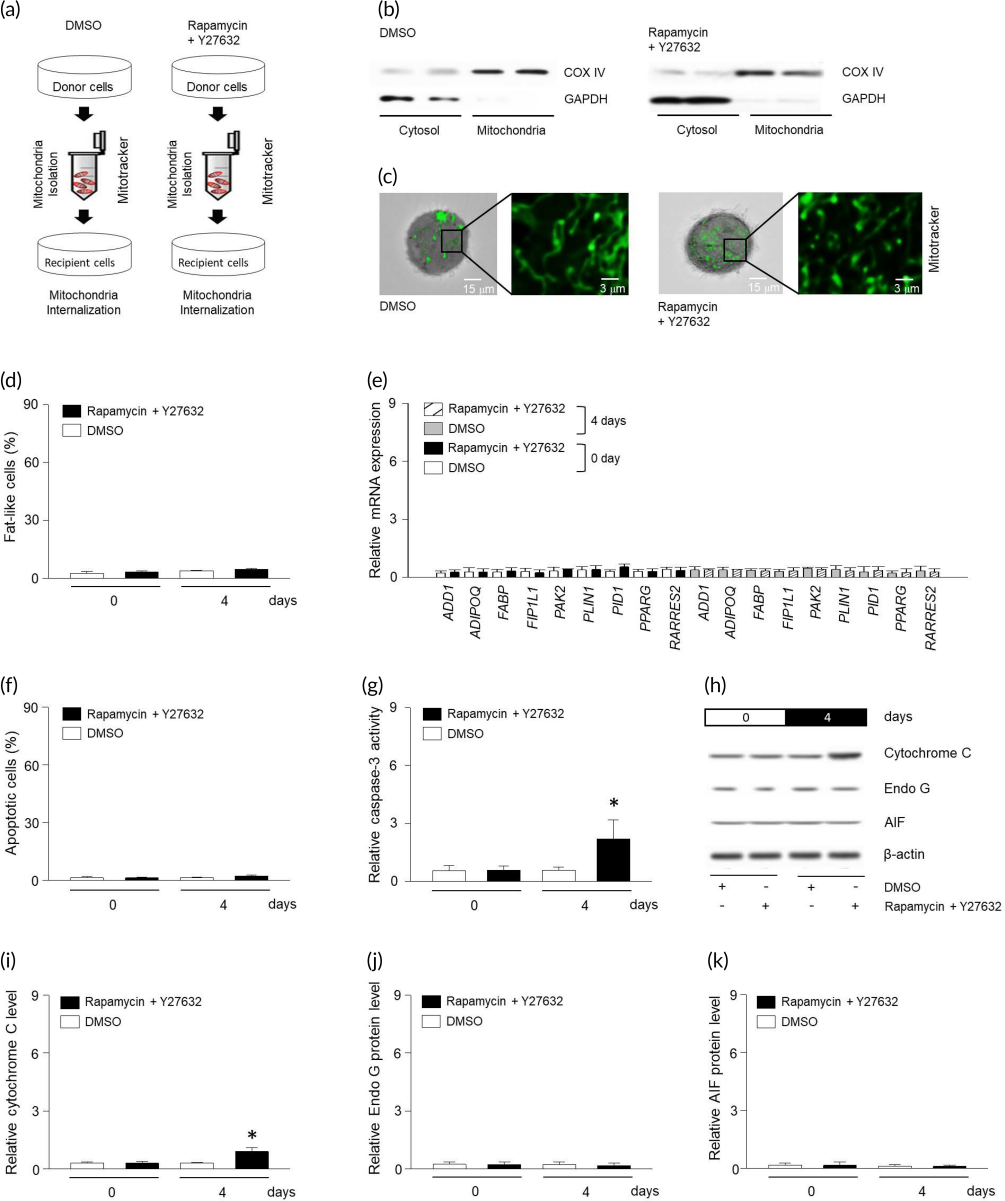
Internalization of mitochondria does not affect differentiation or apoptosis of breast tumor cells. (a) Diagram of mitochondrial isolation and internalization. (b) Western blot for cytosol (GAPDH) and mitochondria fraction (COX IV). (c) Cellular internalization of mitochondria in MDA‐MB‐453 at 4 days. Mitochondria were visualized with mitotracker at 490 nm. (d) Quantization of lipid droplets was determined by tomographic phase microscopy. (e) qRT‐PCR results show relative mRNA levels of defined fat differentiation factors. Apoptosis (f) and caspase‐3 (g) were determined using ELISA kit. (h–k) Proteins were extracted from MDA‐MB‐453 cells and expression of Cytochrome C, Endo G, or AIF was determined using western blotting. Results are the means ± SE of six experiments in each group. Three replicates were performed and the average value was obtained. And then the experiments were repeated on 6 different days. *Significantly different from treatment of rapamycin and Y27632 for 0 days, *p* < 0.05

Migration of mitochondria between cells has been reported to improve viability or metabolism of the recipient cell.^17^, ^18^ However, we hypothesized that the migration induces apoptosis in the recipient cell. To this end, MDA‐MB‐453 cells were treated with ROCK–mTOR inhibitor, and mitochondrial fraction was isolated and confirmed by western blot (Figure 3a,b). The isolated mitochondrial fraction was stained with mitotracker and provided to the tumor cells, which were internalized and were detectable up to 4 days (Figure 3c). Treatment of the isolated mitochondria did not enlarge the number of fat‐like cells, the level of adipocyte markers, or the extent of apoptosis (Figure 3d–f). Interestingly, caspase‐3 activity and cytochrome C were increased following the treatment with the isolated mitochondria (Figure 3g–i); though increased caspase‐3 activation was insufficient to induce tumor cell death. Furthermore, the isolated mitochondria did not alter Endo G or AIF in MDA‐MB‐453 cells (Figure 3j,k).

### 3.4 Physical mimicking of tunneling nanotubes in breast tumor cells

**FIGURE 4:**
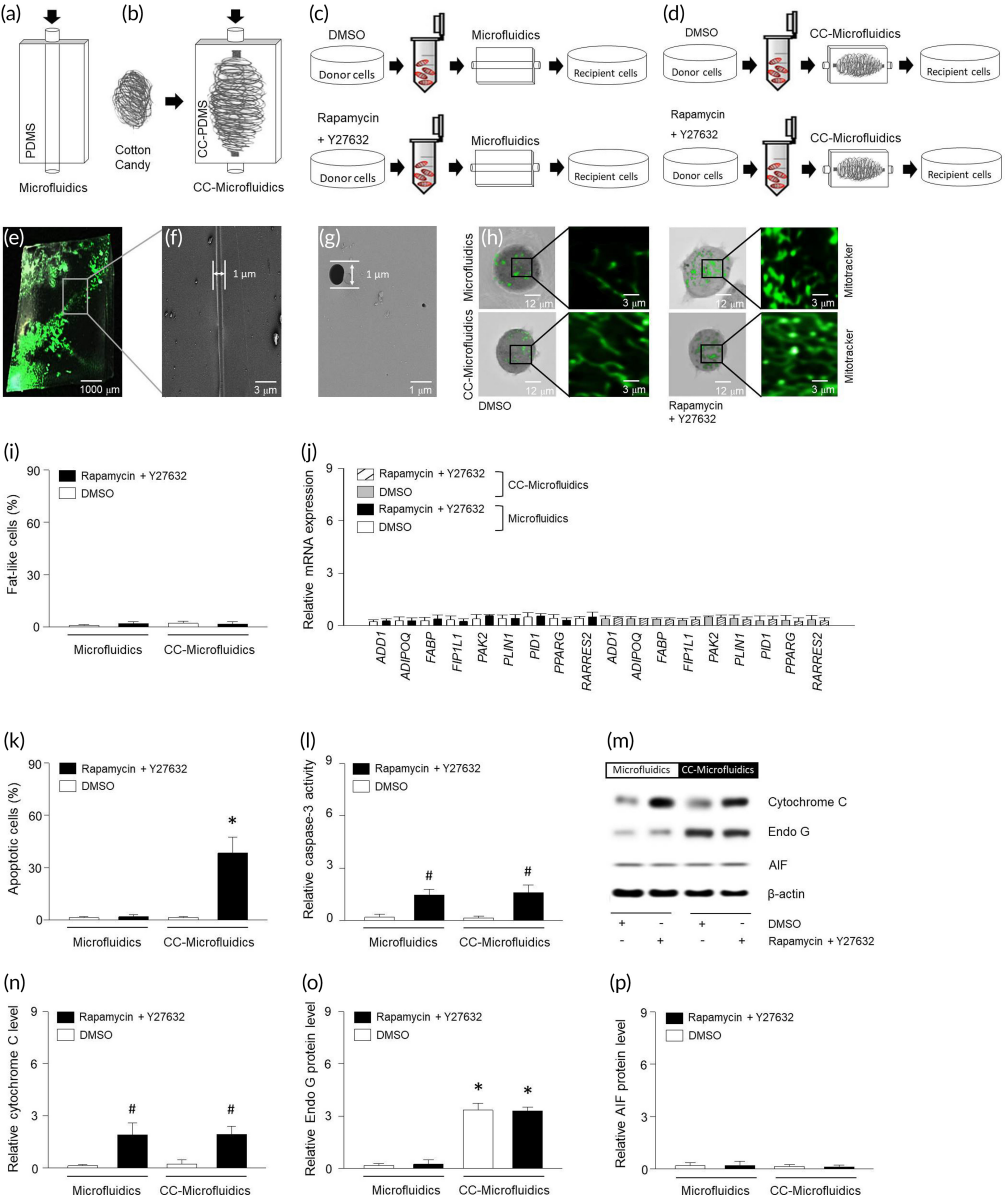
Mitochondria in narrow microfluidic system provoke apoptosis of breast tumor cells. Diagram of a typical microfluidic system (a) with a diameter of 300 μm and a microfluidic mold (B) made by pouring PDMS on cotton candy (CC) with a diameter of around 1 μm, which we referred to herein as CC‐microfluidics. Mitochondria were isolated from DMSO or ROCK inhibitor‐treated cells (MDA‐MB‐453) by centrifugation at 5800*g* for 5 min. The isolated mitochondria were treated into recipient cells (MDA‐MB‐453) after passing through typical microfluidics (C) and CC‐microfluidics (d) for up to 4 days. (e) Macrostructure of CC‐microfluidic illuminated by a 532 nm laser. Transverse (f) or longitudinal (g) cross sections of CC‐microfluidic ultrastructures using electron microscopy. (h) Internalization of mitochondria was visualized with mitotracker at 490 nm. (i) After treating the cells with mitochondria that have passed through two types of microfluidic, lipid droplets were determined by tomographic phase microscopy. (j) qRT‐PCR results show relative mRNA levels of defined fat differentiation factors. Apoptosis (k) and caspase‐3 (l) were determined using ELISA kit. (m–p) Proteins were extracted from MDA‐MB‐453 cells and expression of cytochrome C, Endo G, or AIF were determined using western blotting. Results are the means ± SE of six experiments in each group. Three replicates were performed and the average value was obtained. And then the experiments were repeated on 6 different days. *Significantly different from groups through microfluidic, *p* < 0.05. #Significantly different from treatment of DMSO, *p* < 0.05

In order to mimic the nanotubes through which mitochondria are transferred, we designed a microfluidic system to recapitulate these tunnels. Microfluidic devices were sealed with polydimethylsiloxane (PDMS). The inner channel width was 300 μm and mitochondria were introduced at a flow rate of 10–30 μm/s (Figure 4a). Considering that the diameter of the nanotube is 0.01–1 μm and the mitochondrial diameter is approximately 0.5 μm, PDMS was poured into CC to make a mold, and then water was perfused to remove CC. This resulted in a microfluidic system with a diameter of about 1 μm, which we referred to herein as CC‐microfluidics (Figure 4b). Following treatment with DMSO or ROCK–mTOR inhibitor, mitochondria were stained and isolated from MDA‐MB‐453 cells. The isolated mitochondria were transferred into MDA‐MB‐453 cells via the microfluidic device with a diameter of 300 μm (Figure 4c) or 1 μm (Figure 4d). The CC‐microfluidic device (Figure 4e) was scanned with an electron microscope and the diameter was measured (Figure 4f,g). The internalized mitochondria were present up to 4 days post transfer (Figure 4h). Microfluidic perfusion of the isolated mitochondria did not alter the proportion of fat‐like cells (Figure 4i), adipocyte markers (Figure 4j), or the extent of apoptosis (Figure 4k). On the other hand, caspase activity (Figure 4l) and cytochrome C (Figure 4m,n) were increased compared to DMSO‐treated group. Furthermore, the levels of Endo G or AIF (Figure 4o,p) were not altered. Mitochondrial perfusion using the CC‐microfluidic device also did not alter the proportion of fat‐like cells or levels of adipocyte markers (Figure 4i,j). However, levels of apoptosis (Figure 4k), cleaved caspase 3 (Figure 4l), and cytochrome C (Figure 4m,n) were elevated compared to DMSO‐treated group. Following CC‐microfluidic perfusion, mitochondrial transfer increased Endo G levels in recipient cells, in the presence or absence of ROCK–mTOR inhibitor (Figure 4o). Of note, regardless of chemical treatment, Endo G was increased within the narrow duct with a diameter of about 1 μm.

### 3.5 Endo G is essential for apoptosis through cellular transport of mitochondria

**FIGURE 5:**
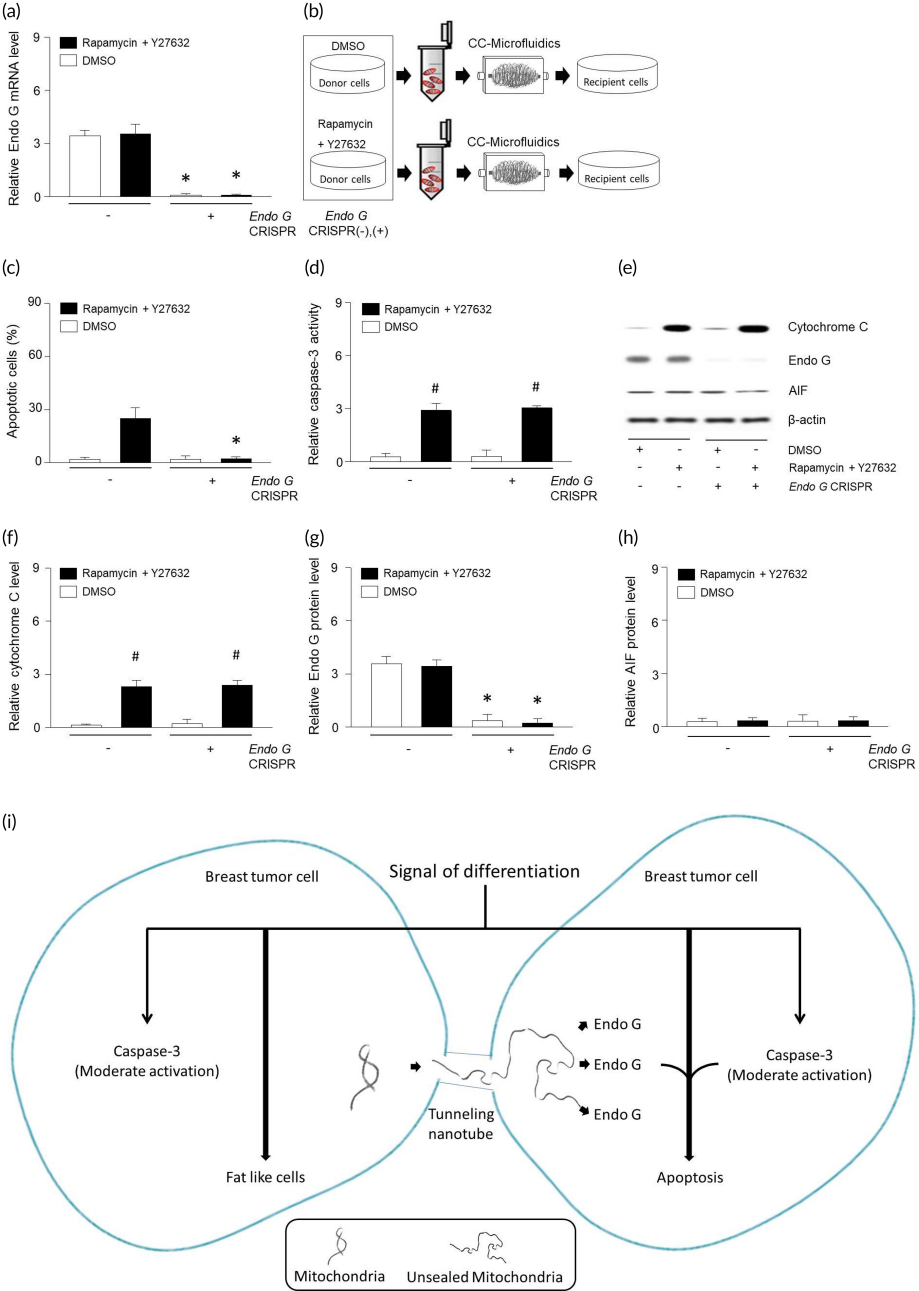
Mitochondria liberated Endo G in narrow microfluidic system. (a) Levels of Endo G miRNA in MDA‐MB‐453 treated with *ENDOG* CRISPR‐plasmid (+) or control CRISPR‐plasmid (−). (b) Mitochondria isolated from CRISPR‐plasmid‐treated cells were passed through CC‐microfluidics and internalized into MDA‐MB‐453. Apoptosis (c) or caspase‐3 (d) was determined using ELISA kit. (e–h) Proteins were extracted from MDA‐MB‐453 cells and expression of cytochrome C, Endo G, or AIF were determined using western blotting. (i) Mitochondria liberated Endo G promoted breast tumor cell apoptosis with moderate caspase‐3 activation through tunneling nanotubes. Results are the means ± SE of six experiments in each group. Three replicates were performed and the average value was obtained. And then the experiments were repeated on 6 different days. *Significantly different from treatment of control CRISPR‐plasmid, *p* < 0.05. #Significantly different from treatment of DMSO, *p* < 0.05

We found that mitochondrial migration through tunneling nanotubes increased the amount of Endo G in the mitochondria‐recipient cells (Figure S2B). In order to better understand the role of Endo G in the context of mitochondria that traveled through CC‐microfluidic perfusion, we knocked down *ENDOG* by CRISPR technology and confirmed the knockdown by real time‐PCR (Figure 5a). Following treatment with DMSO or ROCK–mTOR inhibitor, mitochondria were stained and isolated from MDA‐MB‐453 cells with or without *ENDOG* knockdown (Figure 5b). The CRISPR‐untreated MDA‐MB‐453 was used as recipient cells. Mitochondria transfer from cells deficient in *ENDOG* did not induce apoptosis (Figure 5c), without altering caspase‐3 activity or cytochrome C levels (Figure 5d–f). Furthermore, it did not increase levels of Endo G or AIF of the recipient cells (Figure 5g,h). In caspase‐3‐depleted cells, rapamycin and Y27632 did not induce in apoptosis nor alterations of Endo G levels (Figure S2C–F). Therefore, both caspase‐3 and Endo G are required for apoptosis in this setting. Mitotracker itself did not affect cytochrome C, caspase‐3, Endo G, or apoptosis (Figure S3A–C). The stained and internalized mitochondria were maintained until 4 days, and the loss of mitochondria began to appear after 6 days (Figure S3D). Overall, mitochondrial transport promoted recipient cell apoptosis with release of Endo G through tunneling nanotubes and moderately increased caspase‐3 activity. Mitochondria released Endo G through a narrow space, which we referred to herein as unsealed mitochondria are depicted in a diagram (Figure 5i).

### 3.6 Unsealed mitochondria have a synergistic effect with doxorubicin

**FIGURE 6:**
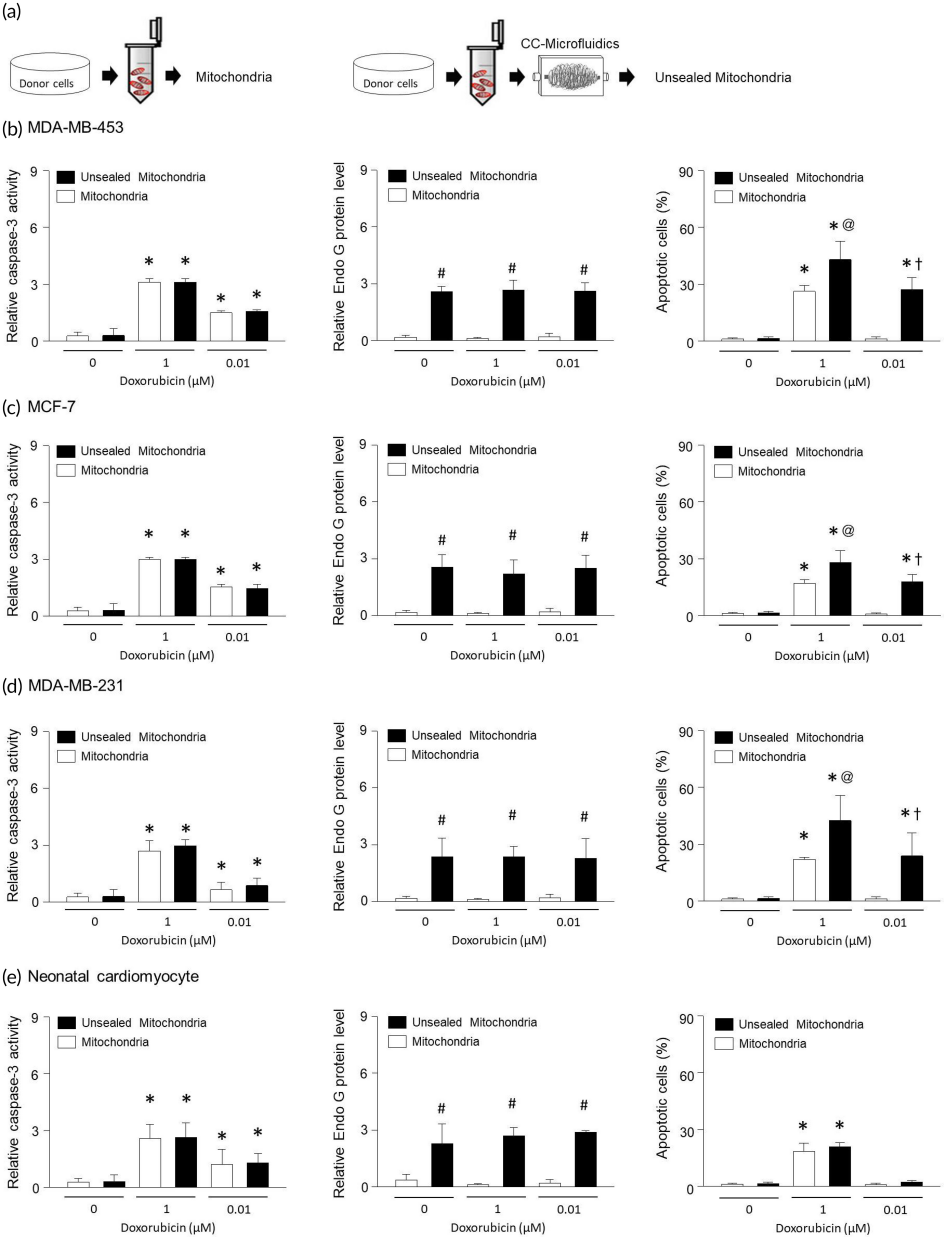
Unsealed mitochondria synergizes with doxorubicin. (a) Diagram of unsealed mitochondrial effects using CC‐microfluidics. Caspase‐3 activation or apoptosis was analyzed using ELISA kit and cytosolic Endo G was determined using western blotting in MDA‐MB‐453 (b), MCF‐7 (c), MDA‐MB‐231 (d), neonatal human cardiomyocytes (e) treated with 1 × 10^4^ unsealed mitochondria, 1 μM or 0.01 μM of doxorubicin for 12 h. Results are the means ± SE of six experiments in each group. Three replicates were performed and the average value was obtained. And then the experiments were repeated on 6 different days. *Significantly different from treatment of 0 μM doxorubicin, *p* < 0.05. #Significantly different from treatment of mitochondria, *p* < 0.05. @Significantly different from treatment of mitochondria with 1 μM doxorubicin, *p* < 0.05. †Significantly different from treatment of mitochondria with 0.01 μM doxorubicin, *p* < 0.05

A working model of the lethal effects of mitochondria is represented in a diagram (Figure 6a). To further assess the lethal effects of mitochondrial transfer, MDA‐MB‐453, MCF‐7, and MDA‐MB‐231 were treated with unsealed mitochondria together with 1 μM of doxorubicin (Figure 6b–d). The activity of caspase‐3 was increased by doxorubicin but did not appear to be dependent on unsealed mitochondria. On the other hand, the release of Endo G was increased by unsealed mitochondria and this did not appear to be affected by doxorubicin. Importantly, unsealed mitochondria induced significant apoptosis combined with low concentration of doxorubicin (0.01 μM), a dose that does not by itself induce apoptosis. Overall, treatment of unsealed mitochondria has a synergistic effect on apoptosis with doxorubicin. Doxorubicin is a widely used chemotherapeutic agent, but it is known to cause cardiotoxicity. Therefore, we investigated the role of unsealed mitochondria on cardiac cells. Human neonatal cardiomyocytes were treated with unsealed mitochondria and doxorubicin; and unsealed mitochondria did not affect caspase‐3 activity in these cells (Figure 6e). Similar to cancer cells, the increased Endo G was dependent on unsealed mitochondria in neonatal cardiomyocyte. However, unlike tumor cells, unsealed mitochondria or unsealed mitochondria with 0.01 μM of doxorubicin did not induce apoptosis in cardiomyocytes. Consistent with these findings, Endo G has been shown to promote cellular survival rather than apoptosis in cardiomyocytes.^23^ In summary, unsealed mitochondria can potentiate lethal effects of doxorubicin in requiring lesser amounts to effectively kill tumor cells in vitro.

### 3.7 Role of unsealed mitochondria in vivo

**FIGURE 7:**
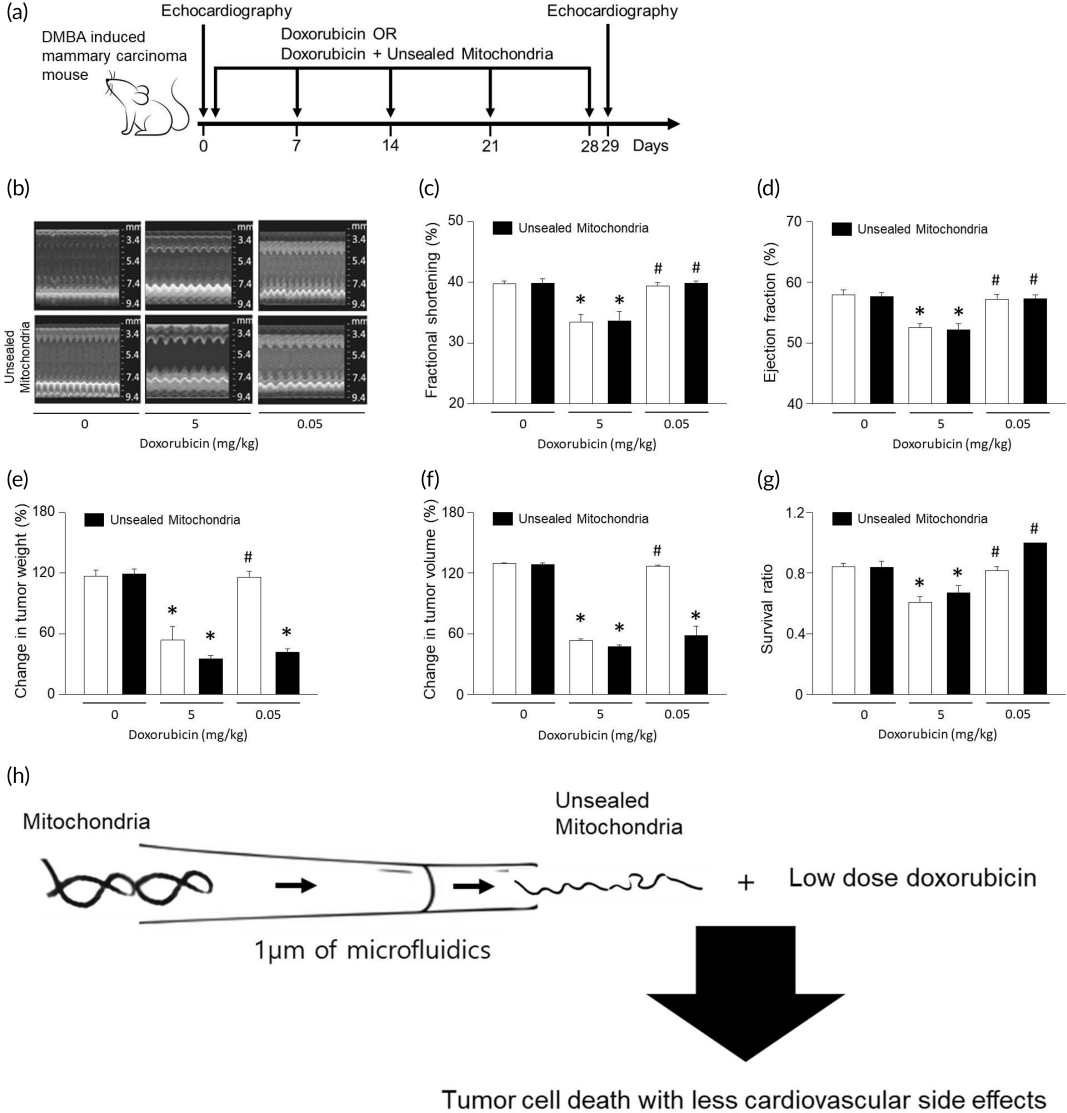
Effect of unsealed mitochondria mice with DMB‐induced mammary carcinoma. (a) Beginning at 5 weeks of age, female mice were given 1 mg of DMBA by oral gavage and 30 mg of subcutaneous pellets of MPA for 6 weeks. Mice with DMBA‐induced mammary carcinoma were treated with doxorubicin or unsealed mitochondria. Doxorubicin was administered weekly by intraperitoneal injection (5 mg/kg or 0.05 mg/kg body weight) for 4 weeks. Unsealed mitochondria were administered weekly (1 × 10^4^/g body weight) via the tail vein. The arrow indicates the injection days. (b) M‐mode echocardiographic images of mice with DMBA‐induced mammary carcinoma treated with doxorubicin or unsealed mitochondria. (c, d) Fractional shortening and ejection fraction were determined from the M‐mode images. (e, f) Tumor volume changes for mice treated with doxorubicin or unsealed mitochondria. (g) Bar graph showing ratio of surviving animals after treatment with doxorubicin or unsealed mitochondria. (h) Unsealed mitochondria synergizes with low doses of doxorubicin in tumoricidal effects in mice. Results are the means ± SE of six experiments in each group. Six mice were used on different days in each experiment. *Significantly different from treatment of 0 mg/kg doxorubicin, *p* < 0.05. #Significantly different from treatment of 0.05 mg/kg doxorubicin, *p* < 0.05

We further assessed the cardiotoxic effects of doxorubicin by echocardiography in mice with DMBA‐induced mammary carcinoma following treatment of doxorubicin with or without unsealed mitochondria (Figure 7a). Fractional shortening and ejection fraction decreased in the group that received 5 mg/kg of doxorubicin (Figure 7b–d). However, the group that received 0.05 mg/kg of doxorubicin alone or doxorubicin with unsealed mitochondria did not result in any contractile dysfunction. Importantly, the low dose (0.05 mg/kg) of doxorubicin together with unsealed mitochondria led to inhibition of tumor growth, whereas this low dose of doxorubicin alone did not exhibit any tumoricidal effects (Figure 7e,f). Consistent with the echocardiographic data, cardiac troponin (cTnT), a specific marker of myocardial damage increased in circulating levels following standard dose of doxorubicin treatment. In contrast, the low dose of doxorubicin did not significantly increase the cTnT (Figure S2A). Therefore, unsealed mitochondria can potentiate doxorubicin, enabling lower noncardiotoxic dose for breast cancer treatment. Unsealed mitochondria also showed a tendency to increase tumor‐free survival rates (Figure 7g). Overall, unsealed mitochondria can effectively induce tumor cell death while avoiding doxorubicin‐induced cardiotoxicity (Figure 7h).

## 4 DISCUSSION

During the reprogramming of breast tumor cells into fat‐like cells, formation of tunneling nanotubes was detected.^3^, ^5^, ^6^ In general, intercellular migration of mitochondria through tunneling nanotubes has been known to impede apoptosis and optimize metabolism.^9^, ^12^, ^15^ However, detection of tunneling nanotubes and mitochondria with accuracy using an optical microscope has had limitations due to suboptimal resolution. Although these components can be visualized with a fluorescence microscope after staining, the difficulties around processing using chemicals and fixatives in addition to the limitations of two‐dimensionality can all contribute to the overall limitations of accurate assessment of tunneling nanotubes and mitochondria. Since tomographic microscopy can provide the internal structure of cell images from multiple angles and render digitally reconstructed images according to the difference in refractive index, the tunneling nanotubes and mitochondria can be distinguished and visualized without the confounding elements that are associated with the processes of special staining.^19^, ^21^ Using such strategies, tomographic images revealed that breast tumor mitochondria migrated to an adjacent cell through tunneling nanotubes and triggered apoptosis. Intriguingly, migration of mitochondria alone did not lead to apoptosis. Therefore, we hypothesized that an additional factor was needed for apoptosis to proceed.

To recapitulate and model a tunneling nanotube, a mold was made by pouring PDMS on CC fibers with a diameter of about 1 μm. Passing through a narrow tube, a significant amount of Endo G was released from mitochondria. However, the Endo G required moderate caspase‐3 activation to induce cell death. In addition, microfluidic system with a diameter of 300 μm did not show any change in Endo G secretion from mitochondria. In order to determine the significance of Endo G in isolated mitochondria, experiments were conducted on breast tumor cells in which *ENDOG* gene was knocked down, and the CRISPR‐untreated MDA‐MB‐453 was used as recipient cells. Mitochondria transfer from cells deficient in *ENDOG* did not induce cell death even though they passed through the microfluidic system with a diameter of 1 μm. In summary, we found that mitochondria released Endo G through the nanotube promoted cell death together with caspase‐3 activation, which we referred to herein as unsealed mitochondria.

To determine the effects of unsealed mitochondria in other breast cancer cell lines, MDA‐MB‐453, MCF‐7, or MDA‐MB‐231 were treated along with doxorubicin. We found that unsealed mitochondria had lethal effects that potentiated the lethal effects of doxorubicin, such that even at low concentrations of doxorubicin, significant apoptosis was achieved when combined with unsealed mitochondria. Importantly, the unsealed mitochondria did not synergize with doxorubicin in inducing apoptosis in cardiomyocytes. Interestingly, in these cells, Endo G was reported to promote cellular survival rather than apoptosis.^23^ Given that doxorubicin is a chemotherapeutic agent known for cardiotoxic effects, the unsealed mitochondria may reduce the off target effects by enabling the use of doxorubicin in low concentration.

In summary, we show that the mitochondria of microfluidics may provide novel strategies to effectively kill tumor cells. Together, we show lethal effects of mitochondria using physical properties of delivery.

## AUTHOR CONTRIBUTIONS

**Hyueyun Kim:** Formal analysis (supporting); investigation (supporting). **Young‐Ho Ahn:** Conceptualization (supporting). **Chang Mo Moon:** Conceptualization (supporting). **Jihee Lee Kang:** Conceptualization (supporting). **Minna Woo:** Writing – review and editing (equal). **Minsuk Kim:** Conceptualization (lead); data curation (lead); formal analysis (lead); funding acquisition (lead); investigation (lead); methodology (lead); project administration (lead); resources (lead); software (lead); supervision (lead); validation (lead); visualization (lead); writing – original draft (lead); writing – review and editing (lead).

## CONFLICT OF INTEREST

The author declares that there is no conflict of interest.

### PEER REVIEW

The peer review history for this article is available at https://publons.com/publon/10.1002/btm2.10461.

## Supporting information


**Supplementary Figure S1.** L‐778123 prevents tunneling nanotubes formation and mitochondria transport
(A) Following treatment of rapamycin (2 μM), Y27632 (2 μM), or L‐778123 (10 μM), MDA‐MB‐453 cells were observed by tomographic microscopy. Mitochondria were stained and visualized with mitotracker at 490 nm. Refractive index images were enlarged at 60, 61, and 62 min. Mitochondria are indicated by arrow heads. (B) Apoptosis was determined using ssDNA ELISA kit. (C) Proteins were extracted and expression of Endo G determined using western blotting. (D) Caspase‐3 activity was determined using ELISA kit. Results are the means ± SE of six experiments in each group. (E) Negative control of mitochondria staining. *Significantly different from treatment of DMSO, *p* < 0.05. #Significantly different from treatment of rapamycin and Y27632, *p* < 0.05.

**Supplementary Figure S2.** Apoptosis of breast tumor requires Endo G and caspase‐3 activity
(A) Plasma level of cardiac troponin was determined using ELISA kit. (B) Following treatment of rapamycin (2 μM) or Y27632 (2 μM), MDA‐MB‐453 cells were observed by tomographic microscopy. Endo G was immunostained and enlarged at 65 min. Endo G was indicated by arrow heads. (C) Levels of caspase‐3 miRNA in MDA‐MB‐453 treated with *CASP3* CRISPR‐plasmid (+) or control CRISPR‐plasmid (−). (D) Apoptosis was determined using ssDNA ELISA kit. (E) Proteins were extracted and expression of Endo G determined using western blotting. (F) Caspase‐3 activity was determined using ELISA kit. Results are the means ± SE of six experiments in each group. *Significantly different from treatment of DMSO, *p* < 0.05. #Significantly different from treatment of rapamycin and Y27632, *p* < 0.05.

**Supplementary Figure S3.** Effects of mitotracker on MDA‐MB‐453 cells
MDA‐MB‐453 treated with *Endo G* CRISPR‐plasmid (+) or control CRISPR‐plasmid (−). Following treatment of mitotracker (1 nM), apoptosis (A) or caspase‐3 (B) activity was determined using ELISA kit. (C) Proteins were extracted and expression of cytochrome C determined using western blotting. (D) The stained and internalized mitochondria were measured by calculating the area of fluorescence for 7 days. Results are the means ± SE of six experiments in each group.

## Data Availability

All data supporting the findings of this study are available within the paper.
