# Supplementary figures and images for: Lethal effects of mitochondria via microfluidics
Source: Bioeng Transl Med. 2022 Dec 5;8(3):e10461. doi: 10.1002/btm2.10461 (PMC10189453; doi:10.1002/btm2.10461)

(A)

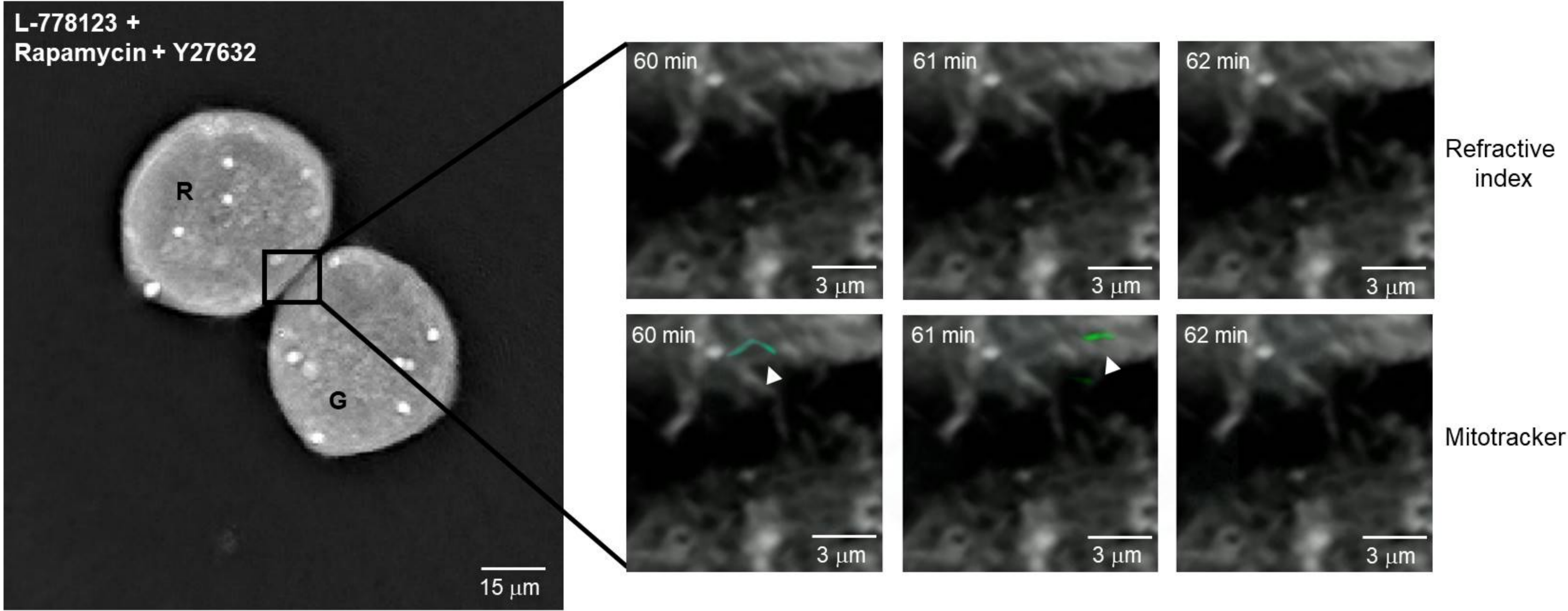

(B)

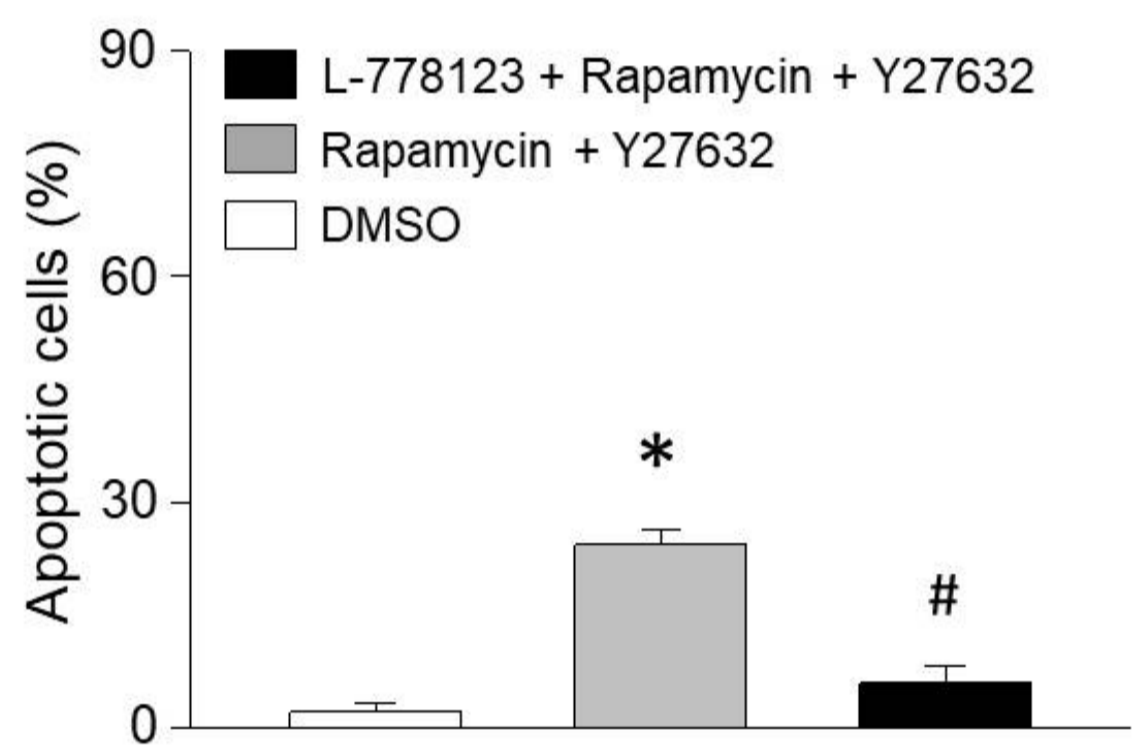

(C)

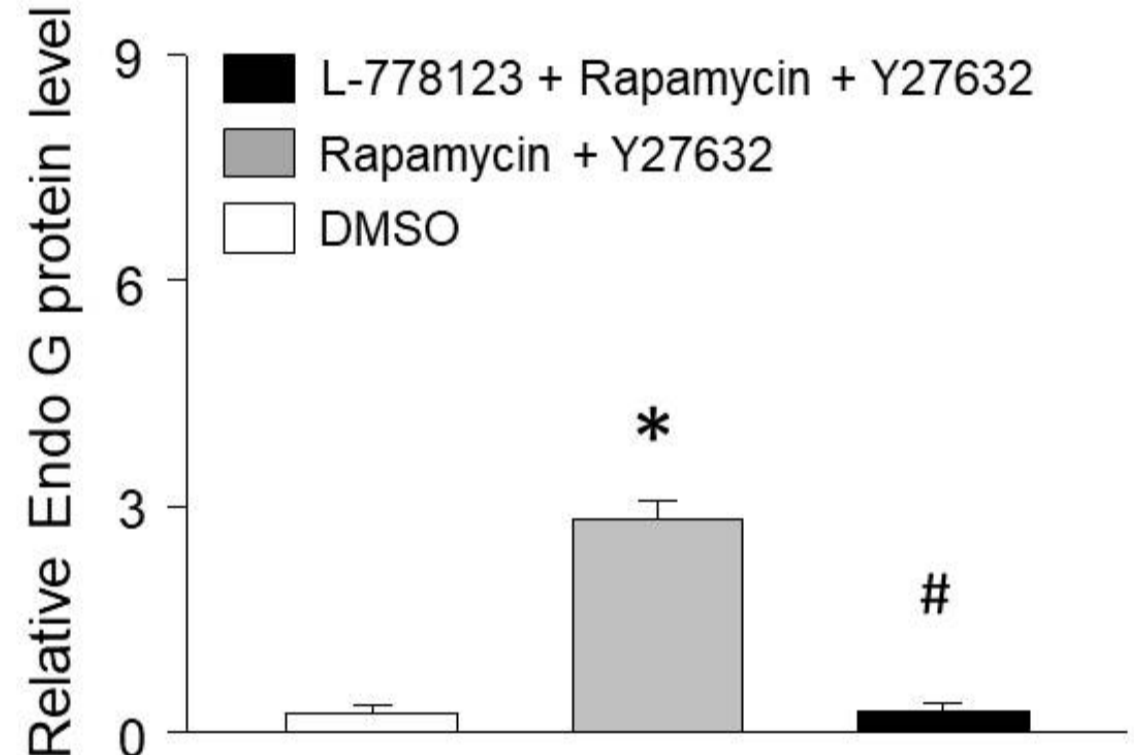

(D)

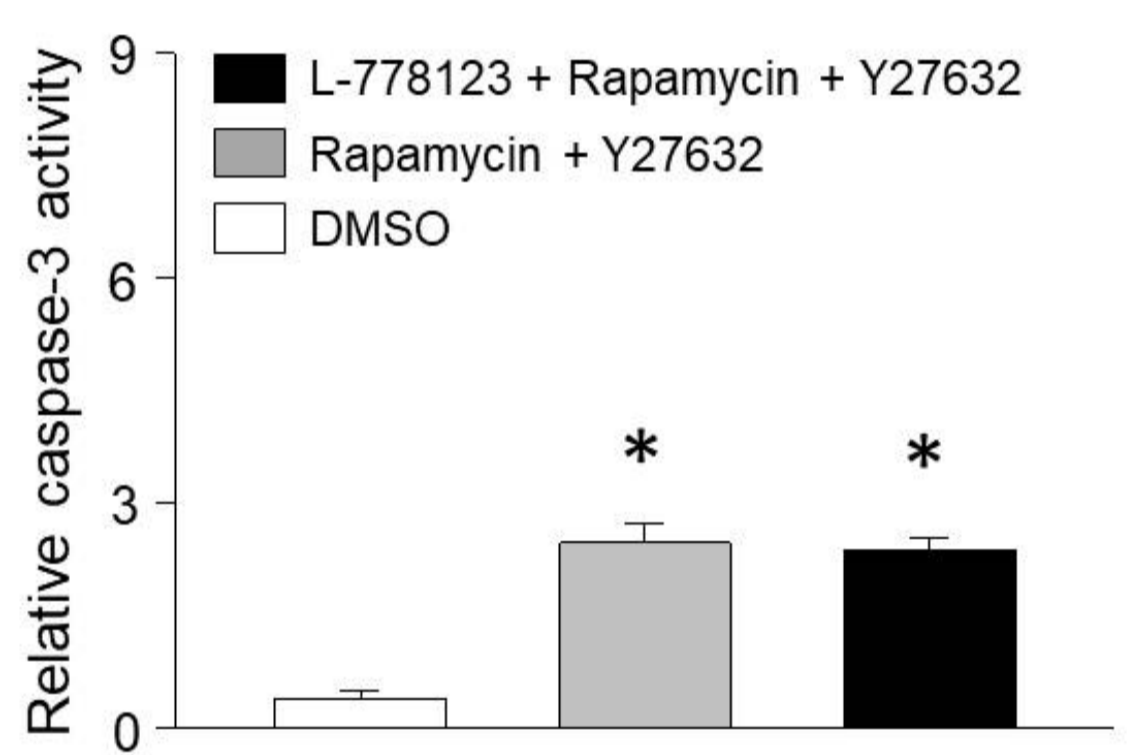

(E)

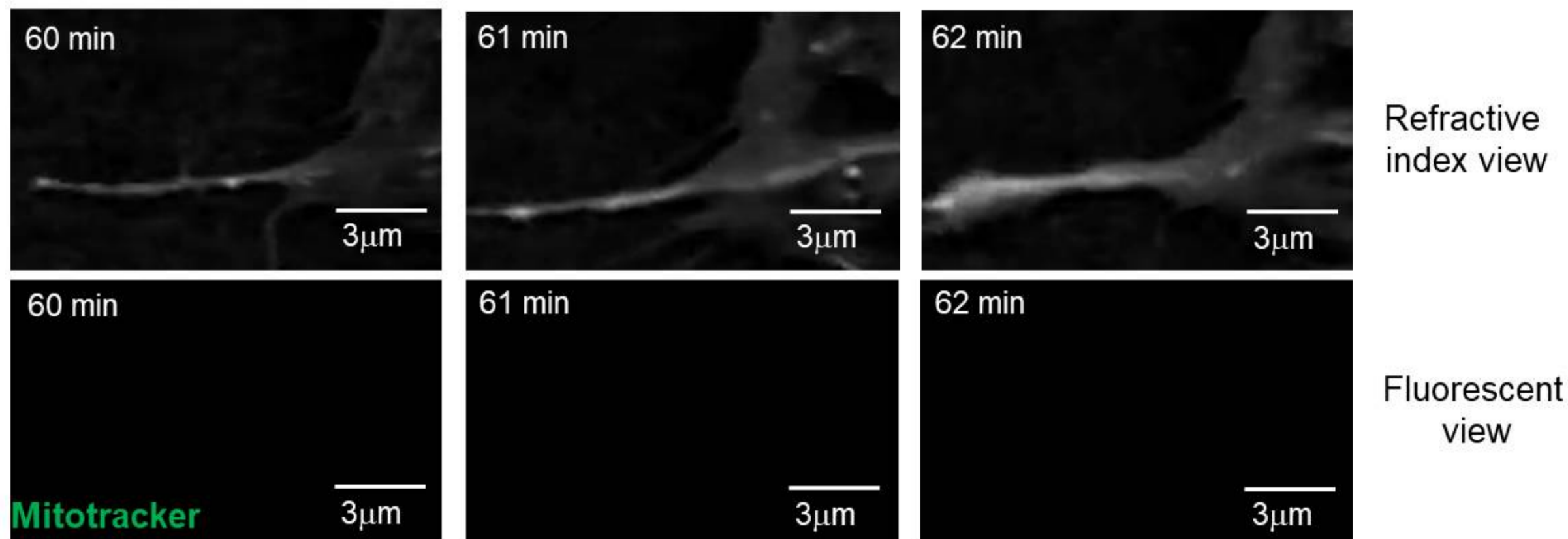

Supplementary Fig. 1

Supplement: Supplementary file 1 — Supplementary Figure S1. L‐778123 prevents tunneling nanotubes formation and mitochondria transport (A) Following treatment of rapamycin (2 μM), Y27632 (2 μM), or L‐778123 (10 μM), MDA‐MB‐453 cells were observed by tomographic microscopy. Mitochondria were stained and visualized with mitotracker at 490 nm. Refractive index images were enlarged at 60, 61, and 62 min. Mitochondria are indicated by arrow heads. (B) Apoptosis was determined using ssDNA ELISA kit. (C) Proteins were extracted and expression of Endo G determined using western blotting. (D) Caspase‐3 activity was determined using ELISA kit. Results are the means ± SE of six experiments in each group. (E) Negative control of mitochondria staining. *Significantly different from treatment of DMSO, p < 0.05. #Significantly different from treatment of rapamycin and Y27632, p < 0.05. [file BTM2-8-e10461-s003.pdf]

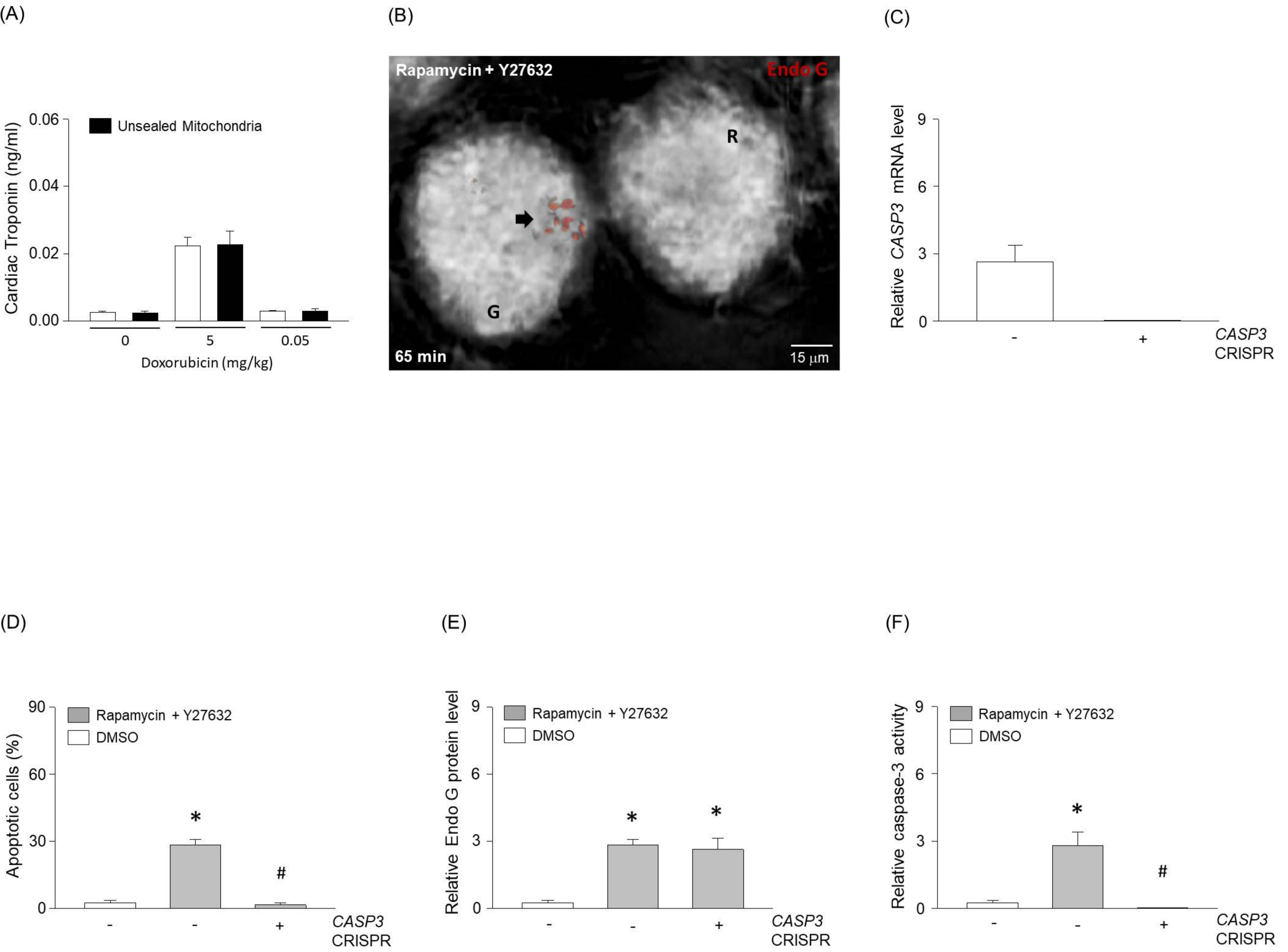

Supplementary Fig. 2

Supplement: Supplementary file 2 — Supplementary Figure S2. Apoptosis of breast tumor requires Endo G and caspase‐3 activity (A) Plasma level of cardiac troponin was determined using ELISA kit. (B) Following treatment of rapamycin (2 μM) or Y27632 (2 μM), MDA‐MB‐453 cells were observed by tomographic microscopy. Endo G was immunostained and enlarged at 65 min. Endo G was indicated by arrow heads. (C) Levels of caspase‐3 miRNA in MDA‐MB‐453 treated with CASP3 CRISPR‐plasmid (+) or control CRISPR‐plasmid (−). (D) Apoptosis was determined using ssDNA ELISA kit. (E) Proteins were extracted and expression of Endo G determined using western blotting. (F) Caspase‐3 activity was determined using ELISA kit. Results are the means ± SE of six experiments in each group. *Significantly different from treatment of DMSO, p < 0.05. #Significantly different from treatment of rapamycin and Y27632, p < 0.05. [file BTM2-8-e10461-s002.pdf]

(A)

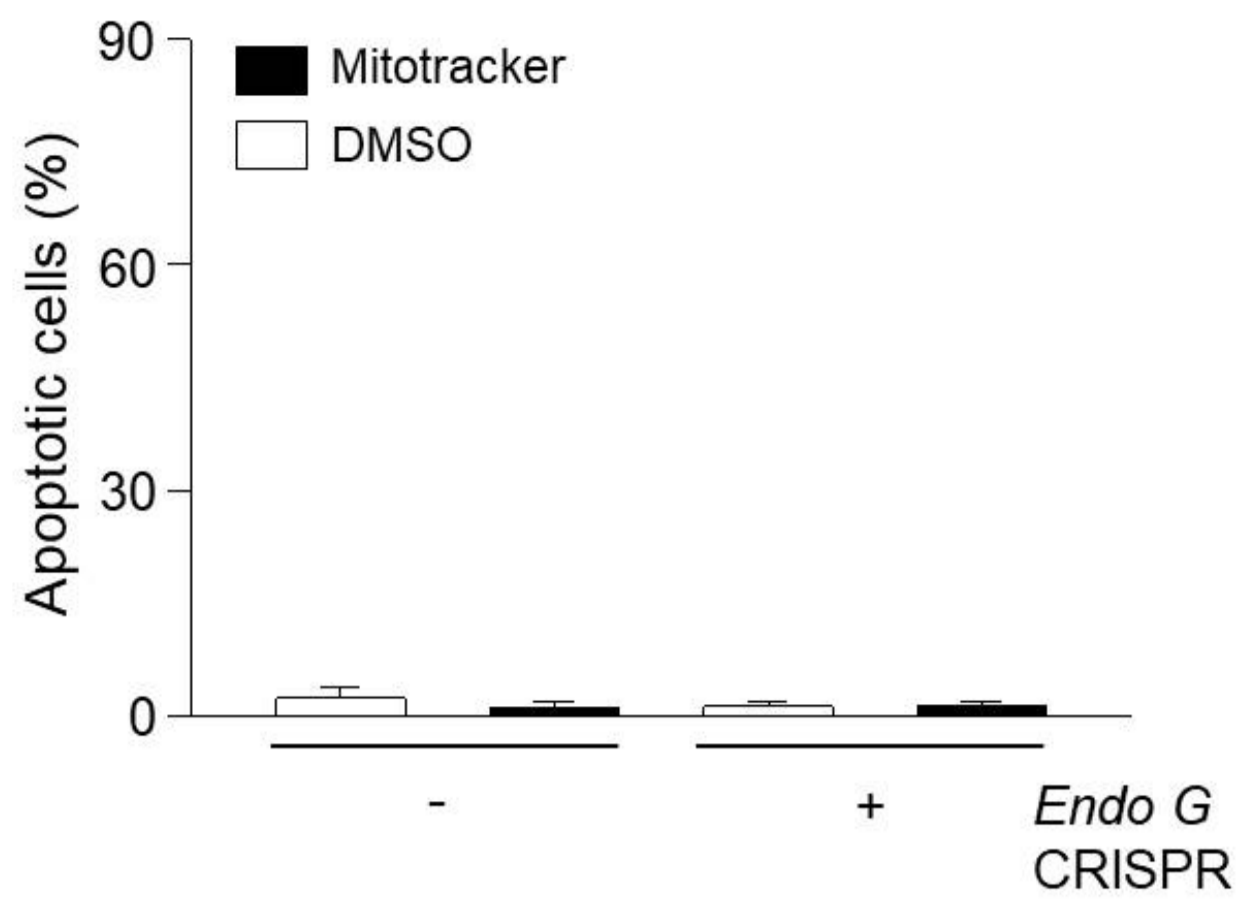

(B)

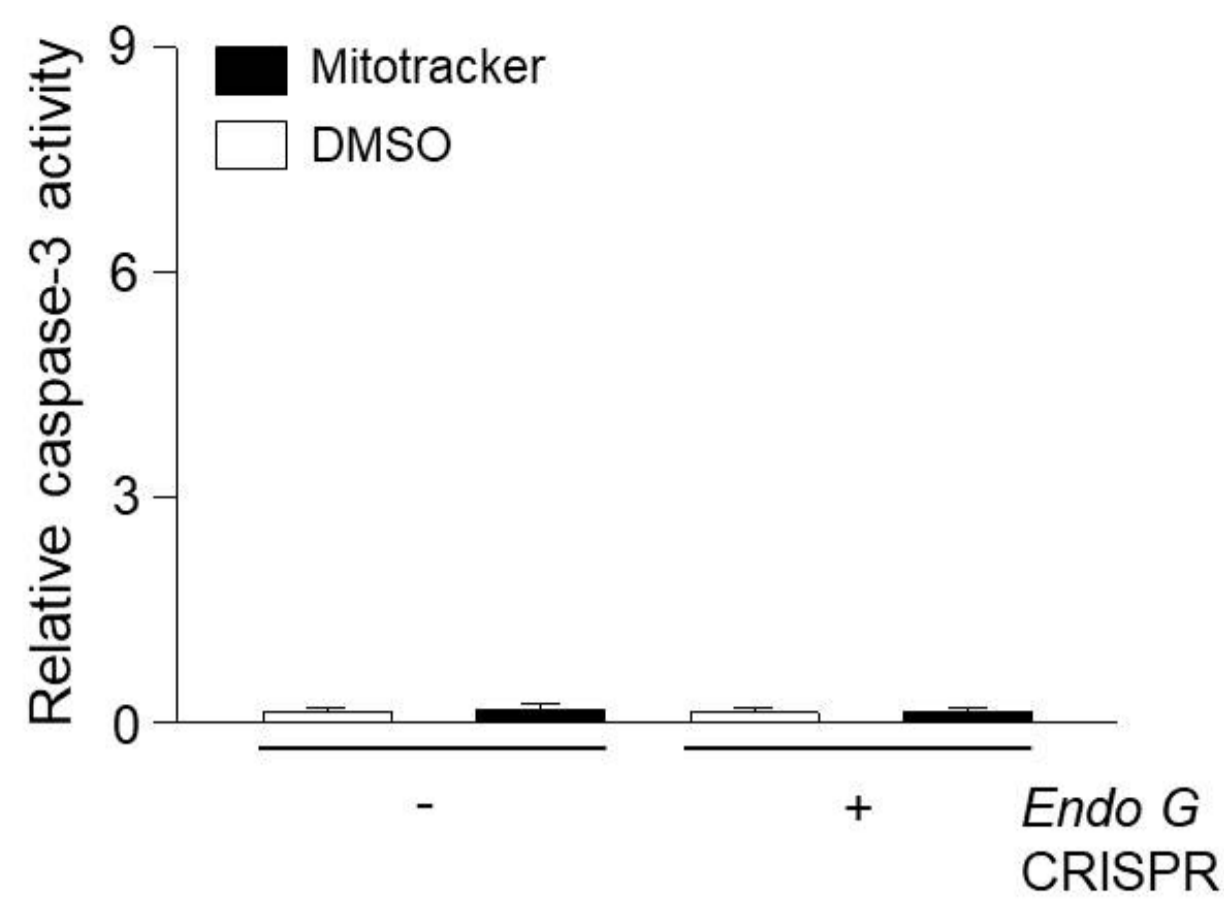

(C)

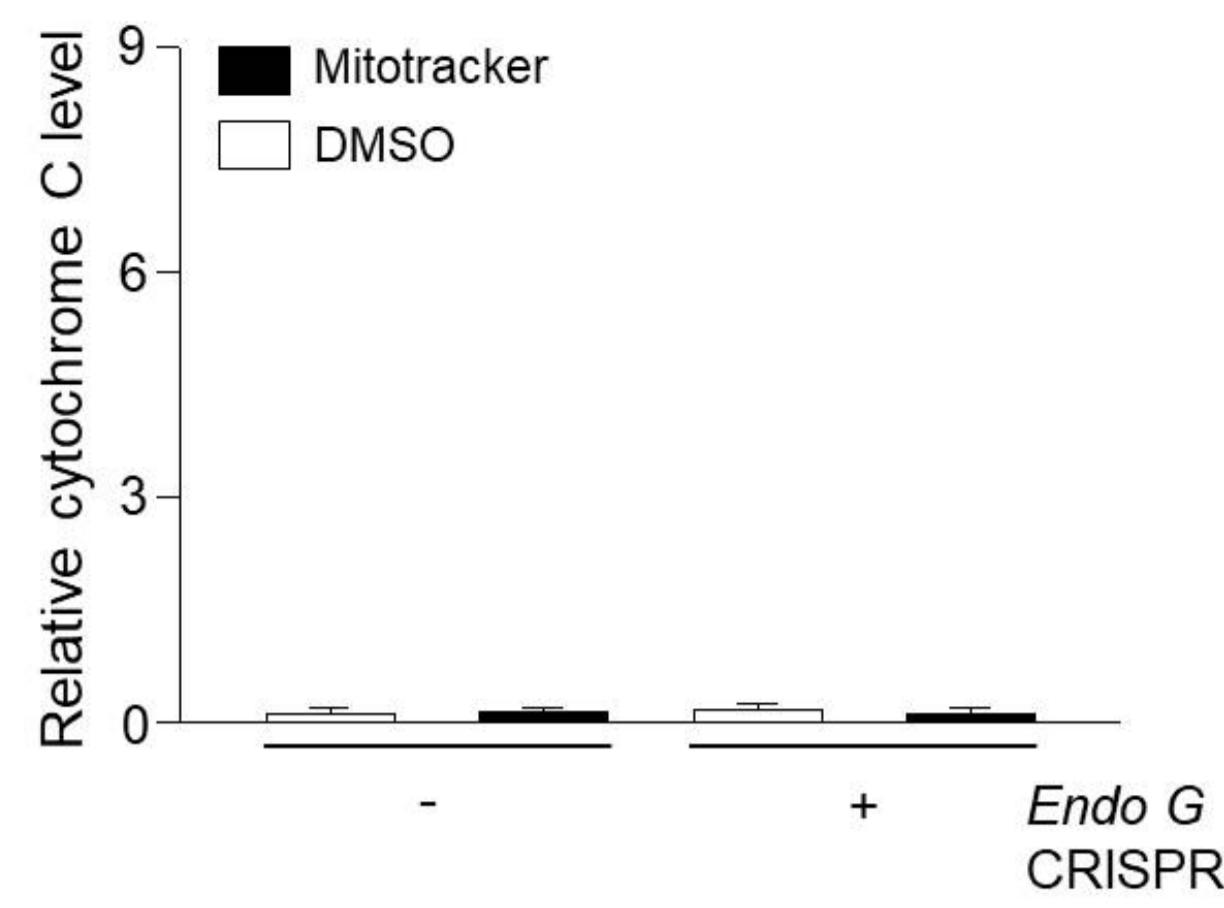

(D)

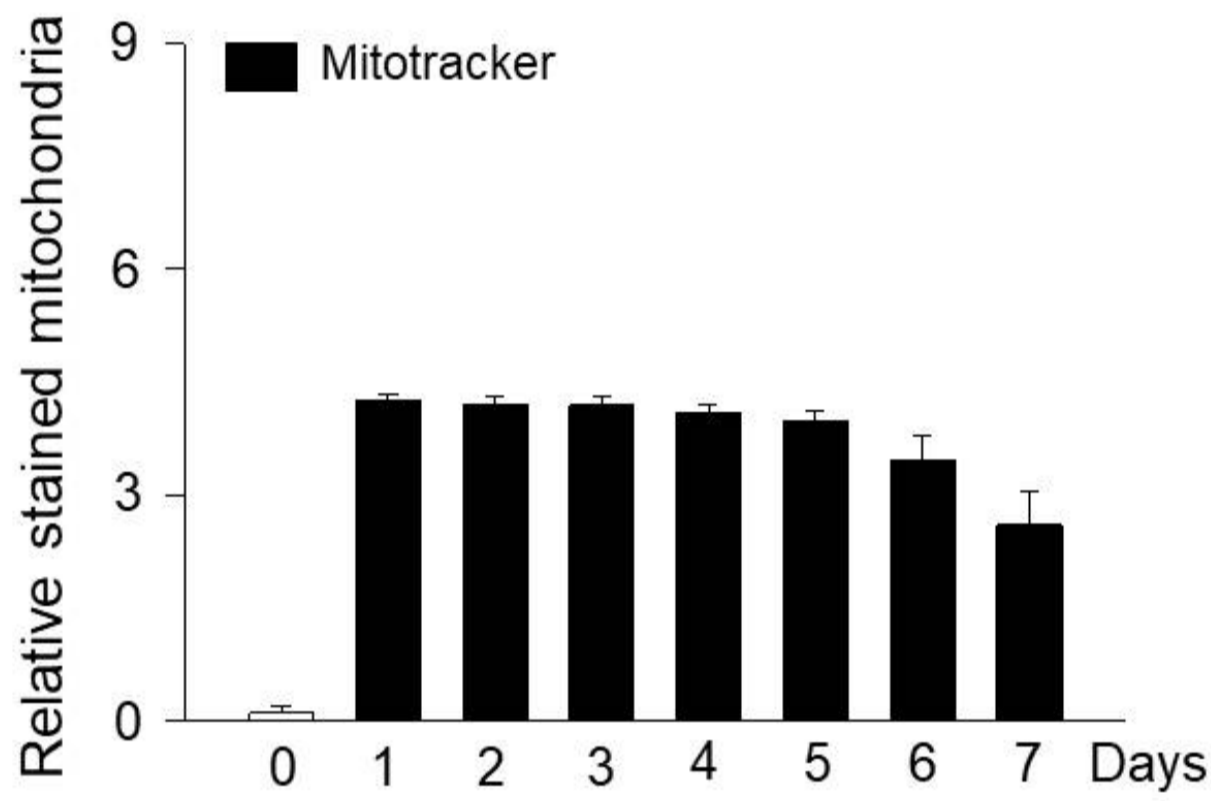

Supplementary Fig. 3

Supplement: Supplementary file 3 — Supplementary Figure S3. Effects of mitotracker on MDA‐MB‐453 cells MDA‐MB‐453 treated with Endo G CRISPR‐plasmid (+) or control CRISPR‐plasmid (−). Following treatment of mitotracker (1 nM), apoptosis (A) or caspase‐3 (B) activity was determined using ELISA kit. (C) Proteins were extracted and expression of cytochrome C determined using western blotting. (D) The stained and internalized mitochondria were measured by calculating the area of fluorescence for 7 days. Results are the means ± SE of six experiments in each group. [file BTM2-8-e10461-s001.pdf]
